# Forensic characteristics and genetic variations of Han Chinese population in Shenzhen revealed by 23 autosomal STRs

**DOI:** 10.3389/fgene.2026.1931015

**Published:** 2026-10-02

**Authors:** Litao Wu, Renhui Jiang, Xinyue Liu, Yinming Zhang

**Affiliations:** 1 Institute of Transfuison Medicine, Shenzhen Blood Center, Shenzhen, China; 2 College of life sciences and oceanography, Shenzhen University, Shenzhen, China

**Keywords:** autosomal short tandem repeats (STR), forensic parameters, genetic variations, paternity testing, Shenzhen Han population

## Abstract

**Introduction:**

Autosomal short tandem repeats (STRs) are essential markers for individual identification and paternity testing. To evaluate their forensic utility in the Shenzhen Han population, we genotyped 1,226 unrelated Shenzhen Han individuals using the Huaxia Platinum PCR kit and obtained allele frequencies for 23 autosomal STR loci. We also calculated forensic parameters, assessed linkage disequilibrium (LD) and Hardy-Weinberg equilibrium (HWE), compared population relationships, and characterized mutation patterns.

**Methods:**

A total of 1,226 unrelated Shenzhen Han individuals were genotyped at 23 autosomal STR loci using the Huaxia Platinum PCR kit. Allele frequencies and forensic parameters were calculated, and LD and HWE were assessed with Bonferroni correction. Population comparisons were performed using seven Chinese and two non-Chinese populations. Mutation patterns were analyzed in 906 trios and 403 duos from 1,309 paternity cases with a combined paternity index 10,000, comprising 2,514 meioses.

**Results:**

No significant HWE deviations were observed after Bonferroni correction. All loci showed high polymorphism and informativeness, with a cumulative power of exclusion (CPE) of 0.999995664709692. Polymorphism information content (PIC) exceeded 0.70 for all loci except CSF1PO, D3S1358, TH01, and TPOX. PentaE (26 alleles) and FGA (23 alleles) were the most polymorphic loci, whereas TPOX (7 alleles), D13S317 (8 alleles), and D16S539 (8 alleles) were the least polymorphic. Population comparisons revealed close genetic affinity between Shenzhen Han and other Chinese groups, especially Hunan Han. Among 2,514 meioses, 30 mutations were observed across the 23 loci, with locus-specific mutation rates ranging from 0 to 0.159% (D5S818) and an overall rate of 0.085% (95% CI, 0.10-2.87 × 10^3^).

**Discussion:**

Our findings confirm that the 23 autosomal STR loci are highly polymorphic and informative in the Shenzhen Han population. These data provide valuable information for forensic and population genetics and support the utility of these loci as a powerful tool for forensic applications.

## Introduction

Short tandem repeat (STR) is abundant and widely distributed in the human genome (Subramanian et al., 2003), numbering over 1.6 million. STR is a kind of DNA regions with a variable number of repeated units (2–6 bps), has a high degree of length polymorphism in the population (Willems et al., 2014; Gymrek et al., 2017). Due to its high information contents, STR has played an important role in population genetics, individual identification (Nwawuba Stanley et al., 2020), paternity testing (Butler, 2007), genomic analyses and research of human evolutionary history (Rowold and Herrera, 2003). The STR loci used in forensic practice originated from 13 STR loci included in the Combined DNA Index System (CODIS). Nowadays, many multiplex amplification techniques can amplify 19 to 29 autosomal STR loci, including these 13 CODIS loci. And we can search existing alleles at various loci in the STRBase database (Ruitberg et al., 2001). In our study, we used the Huaxia Platinum System, which is a 25-loci, six-dye, multiplex that simultaneously amplifies 23 autosomal STR loci (D3S1358, vWA, D16S539, CSF1PO, TPOX, D8S1179, D21S11, D18S51, Penta E, D2S441, D19S433, TH01, FGA, D22S1045, D5S818, D13S317, D7S820, D6S1043, D10S1248, D1S1656, D12S391, D2S1338, and Penta D) as well as gender identification loci of amelogenin and Y-InDel (rs2032678) (Wang et al., 2016; Zhong et al., 2019). Developmental validation demonstrated that Huaxia Platinum System is accurate, sensitive, reproducible and robust, research has suggested that the Huaxia Platinum System is polymorphic and informative, which provides an efficient tool not only for human forensics, but also for population genetics studies, can provide efficient tool for national DNA database and facilitate international data sharing genetics (Wang et al., 2016; Liu et al., 2019; Wang et al., 2018).

Shenzhen is a cosmopolitan city and is one of the administrative districts of Pearl River Delta (PRD), is the second most populated city in Guangdong province, southeast China. It covers a relatively small land area of 1987.005 square kilometers but contains over 17.9895 million residents by the end of 2024. It is bordered by Huizhou to the East, Dongguan to the North, the Pearl River to the West and the Hongkong to the South (Figure 1), which is geographically situated at the crossroads of Chinese inland and the special administrative regions of Hong Kong and Macao. The genetic variants and forensic characteristics, as well as genetic relationships of Shenzhen Han and other nationwide or worldwide populations are largely unknown, thus Shenzhen has been an indispensable piece to understand Chinese population genetic diversity and population structure. However, there are no available genetic data on the Huaxia platinum Kit from the Shenzhen Han population. So our study carried out the genetic analysis of 23 autosomal STR loci in the 1,226 unrelated Han Chinese individuals of Shenzhen area, can expand the available population information for human genetic databases and forensic analysis. Moreover, phylogenetic analysis based on the Nei’s standard genetic distance was performed between the Shenzhen Han population and other relevant populations based on the shared autosomal STR genotyping. Additionally, the genetic polymorphism of STRs was investigated to obtain relatively complete data of STR allele frequency, and to calculate its matching ability (MP), and power of discrimnation (PD), polymorphic information content (PIC), probability of exclusion (PE) and Heterozygosity (Heterozygosity, H) and other forensic genetic parameters to provide basic data for parental identification, individual identification and population genetics in Shenzhen area. To better comprehend the genetic structural background, we compared the studied population with seven other reference Han populations.

**FIGURE 1:**
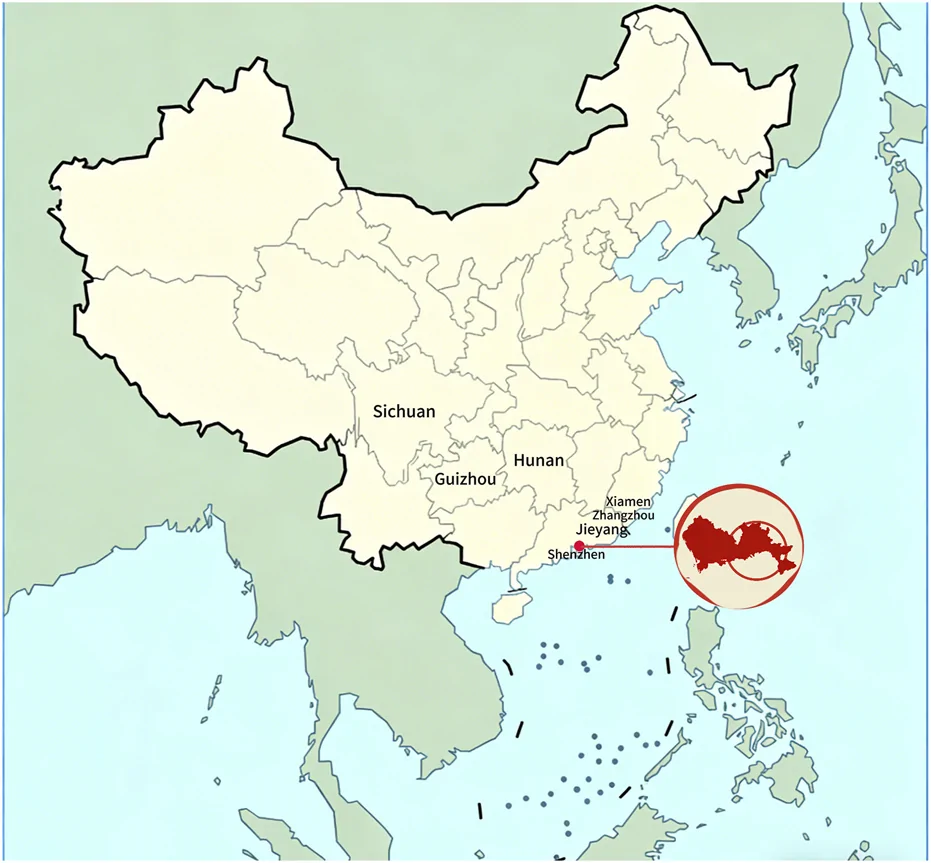
Geographic positions of the Shenzhen Han population and other additional reference Chinese Han populations. The map highlights Shenzhen with a red background.

Mutations of STRs are relatively common in the human genome. As for forensic field, STR mutation can add difficulties to the interpretation of parental testing cases and personal identification (Qian et al., 2015). Although mutation is considered as an important evolutionary force, the high mutation rate of STRs may result in false interpretation of the diverse data and even inappropriate evaluation of genetic evidence in population studies. Therefore mutation studies towards STRs are indispensable and vital, knowledge about the mutation rate of locus-specific STRs is crucial for the correct interpretation of paternity or kinship (Jochens et al., 2011). However, the researches concerning loci in Huaxia Platinum System were primarily concentrated on forensic applications and population genetic analysis, with the mutation analysis rarely being reported. And that was why we chose this kit to be our research subject, attempting to provide the first mutation data and give some insight into mutation rates towards the 23 autosomal STR loci among Shenzhen Han population.

## Materials and methods

### Ethical statement

All participants in this study gave written informed consent, those who did not provide consent were excluded at the onset of our study. The raw data were obtained from routine paternity testing (2019–2025) under a consent clause permitting secondary use of anonymized samples and data for research. To protect participant privacy, all samples and associated demographic data underwent a rigorous de-identification process prior to analysis. Upon collection, direct and indirect identifiers (including names, national identification numbers, dates of birth, and contact details) were removed and replaced with unique, irreversible alphanumeric study codes. The linking key between participant identities and study codes is stored separately on a secure, password-protected institutional server with access limited to the principal investigator.

The study consists of two parts: (1) statistical analysis of existing data, requiring no new experiments; and (2) sequencing analysis of samples with STR mutations from consenting cases. All our work was approved by the Medical Ethics Committee of Shenzhen Blood Center (approval no. SZBCMEC-2025-024).

### Sample collection

STRs were amplified by PCR directly from fingertip puncture using blood collection cards (Changchun Bokun Biotechnology Co., Ltd.) according to the manufacturer’s protocol. A total of 1,226 healthy unrelated individuals were included in the analysis of forensic characteristics, genetic distance. A total of 1,309 paternity testing cases of Shenzhen Han nationality were included in our mutation analysis, with 162 mother-child duos, 241 father-child duos and 906 trios. The true parental relationship was determined by calculating the cumulative paternity index (CPI ≥ 10,000) based on all tested loci.

### PCR amplification and STR typing

23 autosomal STR loci, including the 13 CODIS STR loci were co-amplified in a 10 µL reaction volume using the Huaxia Platinum System (Applied Biosystems, Thermo Fisher Scientific) on GeneAmp® PCR System 9700 (Applied Biosystems, Thermo Fisher Scientific) according to the manufacturer’s recommendations. A total reaction volume of 10 μL comprising 4 μL of reaction mix, 4 μL of primers, 2 μL PnG for samples or 1 μL ddH_2_O and 1 μL of template DNA for positive control. The thermal cycling conditions were as follows: the initial incubation at 95 °C for 1 min; 26 cycles of 94 °C for 3 s, 59 °C for 16 s, 65 °C for 29 s, followed by a final extension step at 65 °C for 15 min. Control DNA and blank blood spot were used as the positive and negative controls, respectively. Capillary electrophoresis and genotyping were performed using a 3500 Series Genetic Analyser (Applied Biosystems, Thermo Fisher Scientific) and sized with GeneScan 600 LIZ Size Standard v2.0 (Thermo Fisher Scientific) on the basis of the user’s manual instructions. 1μL of allelic ladder or PCR products was added to a 10 μL mixture of Hi-Di formamide and size standard. Raw data analysis was performed using GeneMapper ID-X v4.1 Software (Thermo Fisher Scientific).

### Quality control

This work was strictly followed the Guidelines for Autosomal STR Typing by Forensic DNA Testing Laboratories published by Scientific Working Group on DNA Analysis Method (SWGDAM) and was performed in the Shen-Guang forensic authentication institute, Guangdong, which is certificated by China National Accreditation Service for Conformity Assessment Laboratory Accreditation (CNAS).

The positive control DNA and negative control (blank blood spot) were employed for PCR amplification and capillary electrophoresis in each batch of genotyping. All negative controls displayed an absence of amplifed product while positive controls were consistent with known genotypes.

### Statistical analysis

Allele frequencies and forensic parameters, including matching probability (MP), power of discrimination (PD), homozygotes (h), power of exclusion (PE), typical paternity index (TPI), polymorphism information content (PIC), combined match probability (CMP), combined power of discrimination (CPD), and combined power of exclusion (CPE) were evaluated using the Modified PowerStats software Ver 1.2 (Zhao et al., 2003). Arlequin v3.5 (Excoffier and Lischer, 2010) was used to evaluate the expected heterozygosity (He), the observed heterozygosity (Ho) and the *p* value of exact tests of Hardy–Weinberg equilibrium (HWE) and Linkage Disequilibrium (LD). The formulae used to calculate the forensic parameters are listed below:

Observed heterozygosity (Ho): Number of heterozygotes (n)/Number of individuals (N)
Expected heterozygosity (He): $\frac{N}{N-1}\left(1-\sum_{i=1}^{n}{p_{i}}^{2}\right)$

Homozygotes (h): 1-He
Power of exclusion (PE):h^2^*(1–2*h*He^2^)
Typical paternity index (TPI): 1/(2*He)
Power of discrimination (PD): $1-2*{\left(\sum_{i=1}^{n}{p_{i}}^{2}\right)}^{2}+\sum_{i=1}^{n}{p_{i}}^{4}$

Polymorphism information content (PIC): $1-\sum_{i=1}^{n}{p_{i}}^{2}-{\left(\sum_{i=1}^{n}{p_{i}}^{2}\right)}^{2}+\sum_{i=1}^{n}{p_{i}}^{4}$

Matching probability (MP): $\sum_{i=1}^{n}{G_{i}}^{2}$

Genetic distance analysis between Shenzhen Han and another 9 reference populations (Central China, Hunan, Guizhou, Xiamen, Zhangzhou, Jieyang, Sichuan, Thailand, and Malaysia) was calculated by Phylip 3.69 package (Felsenstein, 2009) and the neighbor-joining (N-J) tree construction was applied by the Molecular Evolutionary Genetics Analysis Version 7.0 (Mega 7.0) (Kumar et al., 2016) to further discover the patterns of genetic affinity and reconstruct the phylogenetic relationships. The Principal component analysis (PCA) was performed with the help of OmicShare tools, a free online analysis platform developed by GENE DENOVO (http://www.omicshare.com/tools). The parameters were set by default.

These reference populations were selected according mainly due to geographical location and the inflow sources of the Shenzhen population, such as the Jieyang and Zhangzhou.

The mutation rate for each STR locus was calculated by dividing the total number of allelic transmissions by the number of mutation events, and 95% confidence intervals (CIs) for both the overall and single STR mutation rates were obtained using a website tool (https://statpages.info/confint.html) based on a binomial probability distribution.

### Sequencing

One or two pieces of dried blood spots were punched out and sent to do the sequencing to analyze the mutation characteristics of the STR loci (BGI Genomics Co., Ltd.). DNBSEQ-G99RS High-throughput Sequencing Reagent Kit was used (https://mgi-tech.com/575/44.html). The sequencing platform was DNBSEQ-G99RS (https://mgi-tech.com/fluorescence-emission/6.html), and the sequencing type was SE400.

## Results

### Forensic efficacy

#### STR allele frequencies, Hardy–Weinberg equilibrium (HWE) and forensic parameters

1,226 unrelated Shenzhen Han individuals were successfully genotyped in this study. After applying Bonferroni’s correction (p > 0.0002 = 0.05/253), no prominent discrepancies were observed in linkage disequilibrium (LD) testing (Table 1), indicating the association between the qualitative random variables corresponding to alleles at different STRs. In our study, HWE *p* values for the 23 STR loci ranged from 0.0041 (D7S820) to 0.9924 (D16S539). No deviations from HWE are observed among 23 autosomal STRs after Bonferroni correction of the multiplex test (p = 0.05/23 = 0.0022, where the number of loci is 23 and 0.05 is *p*-value for significance, Table 2). Among the 23 STR loci, TPOX exhibited the lowest allele number 7 whereas the Penta E locus exhibited the highest 26. The allele frequencies for the 23 autosomal STR loci are summarised, with frequencies ranging from 0.0004 (D21S11, 35) to 0.5506 (TPOX, 8) (Table 3).

**TABLE 1:** The *p*-values from Linkage disequilibrium (LD) test for pairs of STR loci.

​	STR Loci	0	1	2	3	4	5	6	7	8	9	10	11	12	13	14	15	16	17	18	19	20	21	22
​	​	CSF1PO	D10S1248	D12S391	D13S317	D16S539	D18S51	D19S433	D1S1656	D21S11	D22S1045	D2S1338	D2S441	D3S1358	D5S818	D6S1043	D7S820	D8S1179	FGA	Penta D	Penta E	TH01	TPOX	vWA
0	CSF1PO	​	​	​	​	​	​	​	​	​	​	​	​	​	​	​	​	​	​	​	​	​	​	​
1	D10S1248	0.51505	​	​	​	​	​	​	​	​	​	​	​	​	​	​	​	​	​	​	​	​	​	​
2	D12S391	0.07545	0.13059	​	​	​	​	​	​	​	​	​	​	​	​	​	​	​	​	​	​	​	​	​
3	D13S317	0.61752	0.89376	0.59069	​	​	​	​	​	​	​	​	​	​	​	​	​	​	​	​	​	​	​	​
4	D16S539	0.56505	0.70535	0.13099	0.89604	​	​	​	​	​	​	​	​	​	​	​	​	​	​	​	​	​	​	​
5	D18S51	0.73178	0.28228	0.64089	0.45376	0.04901	​	​	​	​	​	​	​	​	​	​	​	​	​	​	​	​	​	​
6	D19S433	0.14743	0.40762	0.42317	0.92158	0.7205	0.63446	​	​	​	​	​	​	​	​	​	​	​	​	​	​	​	​	​
7	D1S1656	0.45772	0.3798	0.04604	0.43743	0.47772	0.16564	0.60535	​	​	​	​	​	​	​	​	​	​	​	​	​	​	​	​
8	D21S11	0.35911	0.1298	0.96356	0.1603	0.65792	0.04109	0.49317	0.53059	​	​	​	​	​	​	​	​	​	​	​	​	​	​	​
9	D22S1045	0.62891	0.85921	0.91436	0.27505	0.36198	0.50782	0.42842	0.5695	0.91594	​	​	​	​	​	​	​	​	​	​	​	​	​	​
10	D2S1338	0.32465	0.44337	0.16802	0.82228	0.97238	0.67782	0.37842	0.12178	0.39792	0.33723	​	​	​	​	​	​	​	​	​	​	​	​	​
11	D2S441	0.07228	0.9503	0.63594	0.42376	0.33584	0.49436	0.7101	0.23782	0.19366	0.97832	0.45099	​	​	​	​	​	​	​	​	​	​	​	​
12	D3S1358	0.98386	0.35188	0.29485	0.42535	0.28564	0.04376	0.1605	0.48564	0.54644	0.69178	0.48139	0.05208	​	​	​	​	​	​	​	​	​	​	​
13	D5S818	0.10545	0.01614	0.46416	0.67218	0.7703	0.14059	0.05059	0.55931	0.19347	0.44238	0.89564	0.12614	0.40109	​	​	​	​	​	​	​	​	​	​
14	D6S1043	0.4504	0.35366	0.07772	0.40356	0.74832	0.43842	0.19139	0.81822	0.66059	0.62327	0.34842	0.43426	0.27099	0.12079	​	​	​	​	​	​	​	​	​
15	D7S820	0.53931	0.36594	0.84554	0.62267	0.63149	0.45921	0.71238	0.93812	0.06069	0.8101	0.52515	0.51832	0.3004	0.13119	0.88842	​	​	​	​	​	​	​	​
16	D8S1179	0.85604	0.82762	0.82574	0.42436	0.1703	0.24287	0.38683	0.19693	0.82347	0.0205	0.03851	0.90248	0.90871	0.7495	0.00723	0.41693	​	​	​	​	​	​	​
17	FGA	0.11772	0.09139	0.8297	0.4497	0.99238	0.94317	0.13822	0.97228	0.27554	0.57554	0.86792	0.44366	0.78248	0.03376	0.80564	0.25673	0.69426	​	​	​	​	​	​
18	Penta D	0.06446	0.64574	0.58644	0.96436	0.61168	0.5402	0.04931	0.36673	0.47198	0.91812	0.11871	0.30356	0.32218	0.30554	0.88624	0.09139	0.95178	0.5195	​	​	​	​	​
19	Penta E	0.06515	0.22851	0.14941	0.08851	0.18396	0.43376	0.40594	0.83347	0.72545	0.79416	0.71832	0.08851	0.53089	0.58436	0.75941	0.00406	0.0901	0.85901	0.09693	​	​	​	​
20	TH01	0.50079	0.30604	0.58168	0.10545	0.49624	0.15366	0.30703	0.70535	0.64644	0.18723	0.19812	0.76822	0.13554	0.4901	0.62475	0.32287	0.01921	0.72465	0.01762	0.42881	​	​	​
21	TPOX	0.8095	0.69079	0.20564	0.09545	0.30089	0.31416	0.33356	0.37248	0.95673	0.48307	0.20792	0.35495	0.16743	0.41139	0.39208	0.85861	0.26208	0.16208	0.49386	0.65099	0.96218	​	​
22	vWA	0.7297	0.69653	0.29277	0.5503	0.50614	0.40208	0.27099	0.23574	0.22891	0.8498	0.6996	0.70198	0.55218	0.98406	0.02842	0.63059	0.44505	0.76188	0.02693	0.06644	0.43703	0.34663	​

Significance threshold after Bonferroni correction: *p* = 0.0002.

**TABLE 2:** The *p* values of exact tests of Hardy–Weinberg equilibrium (HWE) for 23 STR in Shenzhen Han population (n = 1,226).

STR loci	*p* values
CSF1PO	0.8242
D10S1248	0.7353
D12S391	0.2827
D13S317	0.7634
D16S539	0.9924
D18S51	0.011
D19S433	0.5312
D1S1656	0.5095
D21S11	0.5798
D22S1045	0.7745
D2S1338	0.9251
D2S441	0.8276
D3S1358	0.0834
D5S818	0.0168
D6S1043	0.7335
D7S820	0.0041
D8S1179	0.3701
FGA	0.9607
Penta D	0.0358
Penta E	0.006
TH01	0.7243
TPOX	0.807
vWA	0.1855

**TABLE 3:** Observed allele frequency distribution and gene diversity in Shenzhen Han population (n = 1,226).

Allele	CSF1PO	D10S1248	D1S1656	D2S441	D2S1338	D3S1358	D5S818	D6S1043	D7S820	D8S1179	D12S391	D13S317	D16S539	D18S51	D19S433	D21S11	D22S1045	FGA	Penta D	Penta E	TH01	TPOX	vWA
2.2	​	​	​	​	​	​	​	​	​	​	​	​	​	​	​	​	​	​	0.0004	​	​	​	​
5	​	​	​	​	​	​	​	​	​	​	​	​	​	​	​	​	​	​	0.0004	0.0518	​	​	​
6	​	​	​	​	​	​	​	​	​	​	​	​	​	​	​	​	​	​	0.0024	​	0.1105	​	​
7	0.0045	​	​	​	​	​	0.0228	​	0.0029	​	​	0.0016	​	​	​	​	​	​	0.0102	0.0012	0.2773	0.0016	​
8	0.0020	0.0029	​	0.0008	​	​	0.0024	​	0.1170	​	​	0.2965	0.0049	​	​	​	​	​	0.0546	0.0029	0.0502	0.5506	​
9	0.0493	​	​	0.0008	​	​	0.0869	0.0016	0.0661	0.0012	​	0.1415	0.2614	​	​	​	​	​	0.3560	0.0114	0.4898	0.1199	​
9.1	​	​	​	0.0261	​	​	​	​	0.0041	​	​	​	​	​	​	​	​	​	​	​	​	​	​
9.3	​	​	​	​	​	​	​	​	​	​	​	​	​	​	​	​	​	​	​	​	0.0302	​	​
10	0.2231	0.0012	0.0041	0.2365	​	​	0.2068	0.0359	0.1660	0.1199	​	0.1334	0.1244	0.0020	0.0004	​	​	​	0.1191	0.0489	0.0420	0.0249	​
10.1	​	​	​	0.0012	​	​	​	​	0.0012	​	​	​	​	​	​	​	​	​	​	​	​	​	​
11	0.2414	0.0029	0.0746	0.3226	​	​	0.3091	0.1060	0.3569	0.1191	​	0.2414	0.2631	0.0033	0.0024	​	0.1921	​	0.1317	0.1607	​	0.2834	​
11.1	​	​	​	0.0004	​	​	​	​	0.0004	​	​	​	​	​	​	​	​	​	​	​	​	​	​
11.2	​	​	​	​	​	​	​	​	​	​	​	​	​	​	0.0004	​	​	​	​	​	​	​	​
11.3	​	​	​	0.0608	​	​	​	​	0.0008	​	​	​	​	​	​	​	​	​	​	​	​	​	​
12	0.3801	0.0722	0.0477	0.1782	​	0.0008	0.2178	0.1272	0.2455	0.1281	​	0.1440	0.2312	0.0400	0.0343	​	0.0004	​	0.1672	0.1170	​	0.0184	​
12.2	​	​	​	​	​	​	​	​	​	​	​	​	​	​	0.0061	​	​	​	​	​	​	​	​
12.3	​	​	​	0.0008	​	​	​	​	​	​	​	​	​	​	​	​	​	​	​	​	​	​	​
13	0.0865	0.3593	0.0922	0.0204	​	0.0020	0.1448	0.1244	0.0355	0.2076	​	0.0347	0.1032	0.1827	0.2896	​	0.0086	0.0008	0.1028	0.0575	​	0.0012	0.0008
13.2	​	​	​	​	​	​	​	​	​	​	​	​	​	​	0.0355	​	​	​	​	​	​	​	​
14	0.0098	0.2520	0.0730	0.1407	​	0.0343	0.0077	0.1570	0.0033	0.1709	​	0.0069	0.0106	0.2194	0.2406	​	0.0232	​	0.0440	0.0905	​	​	0.2798
14.1	​	​	​	0.0004	​	​	​	​	​	​	​	​	​	​	​	​	​	​	​	​	​	​	​
14.2	​	​	​	​	​	​	​	​	​	​	​	​	​	​	0.1069	​	​	​	​	​	​	​	​
15	0.0033	0.2055	0.3083	0.0082	0.0004	0.3238	0.0016	0.0167	0.0004	0.1648	0.0122	​	0.0012	0.1762	0.0730	​	0.3006	​	0.0082	0.0881	​	​	0.0355
15.2	​	​	​	​	​	​	​	​	​	​	​	​	​	​	0.1615	​	​	​	​	0.0004	​	​	​
15.3	​	​	0.0004	​	​	​	​	​	​	​	​	​	​	​	​	​	​	​	​	​	​	​	​
16	​	0.0881	0.2043	0.0020	0.0139	0.3389	​	0.0016	​	0.0730	0.0049	​	​	0.1346	0.0098	​	0.2423	0.0012	0.0029	0.0726	​	​	0.1721
16.1	​	​	0.0008	​	​	​	​	​	​	​	​	​	​	​	​	​	​	​	​	​	​	​	​
16.2	​	​	​	​	​	​	​	​	​	​	​	​	​	0.0004	0.0334	​	​	​	​	​	​	​	​
16.3	​	​	0.0053	​	​	​	​	​	​	​	​	​	​	​	​	​	​	​	​	​	​	​	​
17	​	0.0143	0.0771	​	0.0665	0.2333	​	0.0396	​	0.0131	0.0787	​	​	0.0665	0.0012	​	0.1994	0.0012	​	0.0685	​	​	0.2133
17.2	​	​	​	​	​	​	​	​	​	​	​	​	​	​	0.0045	​	​	​	​	​	​	​	​
17.3	​	​	0.0661	​	​	​	​	0.0004	​	​	​	​	​	​	​	​	​	​	​	​	​	​	​
17.4	​	​	​	​	​	​	​	​	​	​	​	​	​	​	​	​	​	​	​	0.0004	​	​	​
18	​	0.0016	0.0139	​	0.1044	0.0600	​	0.1746	​	0.0024	0.2145	​	​	0.0416	0.0004	​	0.0310	0.0273	​	0.0640	​	​	0.1807
18.2	​	​	​	​	​	​	​	0.0004	​	​	​	​	​	​	​	​	​	​	​	​	​	​	​
18.3	​	​	0.0298	​	​	​	​	​	​	​	​	​	​	​	​	​	​	​	​	​	​	​	​
18.4	​	​	​	​	​	​	​	​	​	​	​	​	​	​	​	​	​	​	​	0.0024	​	​	​
19	​	​	​	​	0.1852	0.0057	​	0.1501	​	​	0.2080	​	​	0.0489	​	​	0.0024	0.0583	​	0.0518	​	​	0.0979
19.3	​	​	0.0020	​	​	​	​	​	​	​	​	​	​	​	​	​	​	​	​	​	​	​	​
19.4	​	​	​	​	​	​	​	​	​	​	​	​	​	​	​	​	​	​	​	0.0012	​	​	​
20	​	​	0.0004	​	0.1244	0.0012	​	0.0498	​	​	0.1852	​	​	0.0232	​	​	​	0.0481	​	0.0522	​	​	0.0179
20.3	​	​	​	​	​	​	​	0.0008	​	​	0.0004	​	​	​	​	​	​	​	​	​	​	​	​
20.4	​	​	​	​	​	​	​	​	​	​	​	​	​	​	​	​	​	​	​	0.0008	​	​	​
21	​	​	​	​	0.0359	​	​	0.0098	​	​	0.1130	​	​	0.0249	​	​	​	0.1228	​	0.0265	​	​	0.0016
21.2	​	​	​	​	​	​	​	​	​	​	​	​	​	​	​	​	​	0.0061	​	​	​	​	​
21.3	​	​	​	​	​	​	​	0.0024	​	​	​	​	​	​	​	​	​	​	​	​	​	​	​
22	​	​	​	​	0.0567	​	​	0.0012	​	​	0.0922	​	​	0.0196	​	​	​	0.1729	​	0.0106	​	​	0.0004
22.2	​	​	​	​	​	​	​	​	​	​	​	​	​	​	​	​	​	0.0029	​	​	​	​	​
22.3	​	​	​	​	​	​	​	0.0004	​	​	​	​	​	​	​	​	​	​	​	​	​	​	​
23	​	​	​	​	0.1852	​	​	​	​	​	0.0522	​	​	0.0118	​	​	​	0.2068	​	0.0098	​	​	​
23.1	​	​	​	​	​	​	​	​	​	​	​	​	​	​	​	​	​	0.0004	​	​	​	​	​
23.2	​	​	​	​	​	​	​	​	​	​	​	​	​	​	​	​	​	0.0069	​	​	​	​	​
24	​	​	​	​	0.1578	​	​	​	​	​	0.0228	​	​	0.0037	​	​	​	0.1746	​	0.0049	​	​	​
24.2	​	​	​	​	​	​	​	​	​	​	​	​	​	​	​	​	​	0.0082	​	​	​	​	​
25	​	​	​	​	0.0559	​	​	​	​	​	0.0131	​	​	0.0004	​	​	​	0.0901	​	0.0033	​	​	​
25.2	​	​	​	​	​	​	​	​	​	​	​	​	​	​	​	​	​	0.0016	​	​	​	​	​
26	​	​	​	​	0.0126	​	​	​	​	​	0.0020	​	​	0.0004	​	0.0004	​	0.0493	​	0.0004	​	​	​
26.2	​	​	​	​	​	​	​	​	​	​	​	​	​	​	​	​	​	0.0024	​	​	​	​	​
27	​	​	​	​	0.0012	​	​	​	​	​	0.0008	​	​	​	​	0.0008	​	0.0151	​	​	​	​	​
27.2	​	​	​	​	​	​	​	​	​	​	​	​	​	​	​	​	​	0.0004	​	​	​	​	​
28	​	​	​	​	​	​	​	​	​	​	​	​	​	0.0004	​	0.0542	​	0.0016	​	​	​	​	​
28.2	​	​	​	​	​	​	​	​	​	​	​	​	​	​	​	0.0041	​	​	​	​	​	​	​
29	​	​	​	​	​	​	​	​	​	​	​	​	​	​	​	0.2692	​	0.0008	​	​	​	​	​
29.2	​	​	​	​	​	​	​	​	​	​	​	​	​	​	​	0.0004	​	​	​	​	​	​	​
30	​	​	​	​	​	​	​	​	​	​	​	​	​	​	​	0.2529	​	​	​	​	​	​	​
30.2	​	​	​	​	​	​	​	​	​	​	​	​	​	​	​	0.0090	​	​	​	​	​	​	​
30.3	​	​	​	​	​	​	​	​	​	​	​	​	​	​	​	0.0012	​	​	​	​	​	​	​
31	​	​	​	​	​	​	​	​	​	​	​	​	​	​	​	0.0942	​	​	​	​	​	​	​
31.2	​	​	​	​	​	​	​	​	​	​	​	​	​	​	​	0.0685	​	​	​	​	​	​	​
32	​	​	​	​	​	​	​	​	​	​	​	​	​	​	​	0.0306	​	​	​	​	​	​	​
32.2	​	​	​	​	​	​	​	​	​	​	​	​	​	​	​	0.1489	​	​	​	​	​	​	​
33	​	​	​	​	​	​	​	​	​	​	​	​	​	​	​	0.0041	​	​	​	​	​	​	​
33.2	​	​	​	​	​	​	​	​	​	​	​	​	​	​	​	0.0563	​	​	​	​	​	​	​
34	​	​	​	​	​	​	​	​	​	​	​	​	​	​	​	0.0012	​	​	​	​	​	​	​
34.2	​	​	​	​	​	​	​	​	​	​	​	​	​	​	​	0.0037	​	​	​	​	​	​	​
35	​	​	​	​	​	​	​	​	​	​	​	​	​	​	​	0.0004	​	​	​	​	​	​	​

PIC in Shenzhen Han Chinese population varies from 0.5407 (TPOX) to 0.9096 (PentaE) and all 23 studied STRs are observed to be fairly informative (PIC>0.5). The locus of Penta E is found to be the most informative, followed by D6S1043 (0.8605) and D2S1338 (0.8536), indicating that the 23 autosomal STR genetic markers included in the Huaxia Platinum Kit are suitable for genetic characterisation and diversity studies.

Heterozygosity (H) is one of the key factors for studying genetic variation in natural populations. He and Ho span from 0.6101 (TPOX) to 0.9119 (PentaE), and from 0.6014 (TPOX) to 0.9160 (Penta E), respectively. The matching probability (MP) measures the probability of an unrelated person in a population and the lower the value of MP the more informative that particular STR will be. MP and PD vary from 0.0137 (PentaE) to 0.0222 (TPOX), and 0.7784 (TPOX) to 0.9863 (PentaE), respectively. The paternity PE is the expected average probability that a polymorphic locus shows the exclusion of a man without kinship with the biological father. This index depends on the informative content of a locus, which depends on its number of alleles and its respective frequencies. PE ranges from 0.3032 (TPOX) to 0.8198 (PentaE), and TPI spans from 1.282 (TPOX) to 5.6759(PentaE). The combined probability of match is 3.0911 × 10^−28^, and the combined probability of exclusion (CPE) is 0.999995664709692 (Table 4). Our results illustrated that STR loci included in the Huaxia Platinum System are high diversity and can be used as a powerful tool in forensic individual identification and parentage testing in the Shenzhen Han population.

**TABLE 4:** Forensic statistics for 23 autosomal STR loci in Shenzhen Han population (n = 1,226).

Allele	CSF1PO	D10S1248	D12S391	D13S317	D16S539	D18S51	D19S433	D1S1656	D21S11	D22S1045	D2S1338	D2S441	D3S1358	D5S818	D6S1043	D7S820	D8S1179	FGA	Penta D	Penta E	TH01	TPOX	vWA
MP	0.1107	0.1023	0.0422	0.0739	0.0812	0.0361	0.0608	0.0465	0.0542	0.0881	0.0327	0.0791	0.1302	0.0784	0.0296	0.0909	0.0411	0.0328	0.0688	0.0137	0.1637	0.2216	0.0676
PD	0.8893	0.8977	0.9578	0.9261	0.9188	0.9639	0.9392	0.9535	0.9458	0.9119	0.9673	0.9209	0.8698	0.9216	0.9704	0.9091	0.9589	0.9672	0.9312	0.9863	0.8363	0.7784	0.9324
PIC	0.6953	0.7130	0.8269	0.7639	0.7482	0.8419	0.7875	0.8122	0.7985	0.7356	0.8536	0.7521	0.6704	0.7527	0.8605	0.7313	0.8318	0.8500	0.7748	0.9095	0.6177	0.5407	0.7742
PE	0.4695	0.5431	0.6692	0.6161	0.5577	0.6915	0.6315	0.6192	0.6146	0.5504	0.7418	0.5547	0.4655	0.5562	0.7287	0.5562	0.6931	0.7206	0.6253	0.8198	0.4045	0.3032	0.5963
TPI	1.8244	2.1661	3.0650	2.6197	2.2454	3.2957	2.7366	2.6422	2.6085	2.2050	3.9548	2.2291	1.8083	2.2372	3.7607	2.2372	3.3135	3.6488	2.6886	5.6759	1.5840	1.2824	2.4818
H	0.2741	0.2308	0.1631	0.1909	0.2227	0.1517	0.1827	0.1892	0.1917	0.2268	0.1264	0.2243	0.2765	0.2235	0.1330	0.2235	0.1509	0.1370	0.1860	0.0881	0.3157	0.3899	0.2015
He	0.7259	0.7692	0.8369	0.8091	0.7773	0.8483	0.8173	0.8108	0.8083	0.7732	0.8736	0.7757	0.7235	0.7765	0.8670	0.7765	0.8491	0.8630	0.8140	0.9119	0.6843	0.6101	0.7985
Ho	0.7377	0.7522	0.8457	0.7943	0.7831	0.8578	0.8121	0.8304	0.8210	0.7730	0.8681	0.7839	0.7214	0.7855	0.8741	0.7658	0.8504	0.8647	0.7984	0.9160	0.6661	0.6014	0.8031

MP: the observed Matching Probability; PD: power of discrimination; PIC: polymorphic information content; PE: the mean Probability of exclusion; TPI: typical paternity index; h: Homozygotes; He: Expected heterozygosity; Ho: Observed heterozygosity.

### Population comparisons

To investigate the genetic structure of the Shenzhen Han population and its genetic relationships with other Chinese Han populations, we performed a series of analyses based on the genotype data of the Huaxia Platinum System among 9 populations including central China (Hongdan et al., 2017), Xiamen (Lu et al., 2017), Zhangzhou (Wu et al., 2020), Jieyang, Guizhou (Zhang et al., 2015), Sichuan (H et al., 2018), Hunan, Thialand, Malaysia (Hakim et al., 2020). Phylogenetic relationship among 9 populations is constructed on the basis of Fst genetic matrix and N-J algorithm, and the Fst pairwise genetic distances are presented (Table 5). Although the Shenzhen group is geographically closer to populations such as Hunan, Guizhou, Xiamen, Zhangzhou, Jieyang, and Sichuan, the Neighbor-joining tree indicates that the genetic relationship between the Han population in Shenzhen and Hunan is closer than that between Shenzhen and other groups.The groups from Xiamen, Zhangzhou, Jieyang, and so on are genetically more distant from the Han population in Shenzhen (Figure 2). PCA (Principal Component Analysis) results based on the allele frequencies of the 23 shared STR loci are illustrated (Table 6; Figure 3). PCA captured approximately 82.15% of the genetic variations among 10 populations, with the first and second component accounting for 62.15% and 20%, respectively. Based on the first principal component, samples exhibited a clear geographic differentiation: all Chinese samples (including Shenzhen, Central China, Hunan, Guizhou, Xiamen, Zhangzhou, Jieyang, and Sichuan) were distributed on the negative side, with PC1 scores highly clustered and ranging only from −0.132 (Central China) to −0.048 (Shenzhen). The extremely close distances among them suggest a high degree of homogeneity in the compositional features represented by PC1. In stark contrast, Thailand (0.320) and Malaysia (0.264) both displayed large positive PC1 scores, clearly deviating from the Chinese sample cluster, indicating that PC1 strictly separates the “China” region from the “Southeast Asia” region. Based on the second principal component, further separation was observed mainly within Southeast Asia: Thailand had the highest PC2 score (0.165), while Malaysia had the lowest (−0.201), showing a bipolar distribution along PC2. This implies that, despite both being in Southeast Asia, the two countries differ substantially in the characteristics captured by this second dimension. In comparison, all Chinese samples had PC2 scores concentrated between −0.034 (Central China) and 0.030 (Guizhou), remaining tightly clustered on the PC2 axis with no prominent outliers.

**TABLE 5:** Genetic distance analysis between the population in Shenzhen and nine other populations.

Populations	Shenzhen	Central China	Hunan	Guizhou	Xiamen	Zhangzhou	Jieyang	Sichuan	Thailand	Malaysia
Shenzhen	​	​	​	​	​	​	​	​	​	
Central China	0.005687	​	​	​	​	​	​	​	​	
Hunan	0.002715	0.005433	​	​	​	​	​	​	​	
Guizhou	0.002904	0.005985	0.002892	​	​	​	​	​	​	
Xiamen	0.001372	0.003887	0.00215	0.002545	​	​	​	​	​	
Zhangzhou	0.001729	0.004689	0.002514	0.003269	0.000843	​	​	​	​	
Jieyang	0.003856	0.006373	0.00459	0.005539	0.00199	0.00255	​	​	​	
Sichuan	0.00161	0.00399	0.002109	0.001236	0.001116	0.001667	0.004004	​	​	
Thailand	0.026755	0.041017	0.028573	0.029441	0.029052	0.030256	0.030979	0.029141	​	
Malaysia	0.024754	0.032396	0.025775	0.027791	0.024568	0.025908	0.026355	0.025428	0.022617	

**FIGURE 2:**
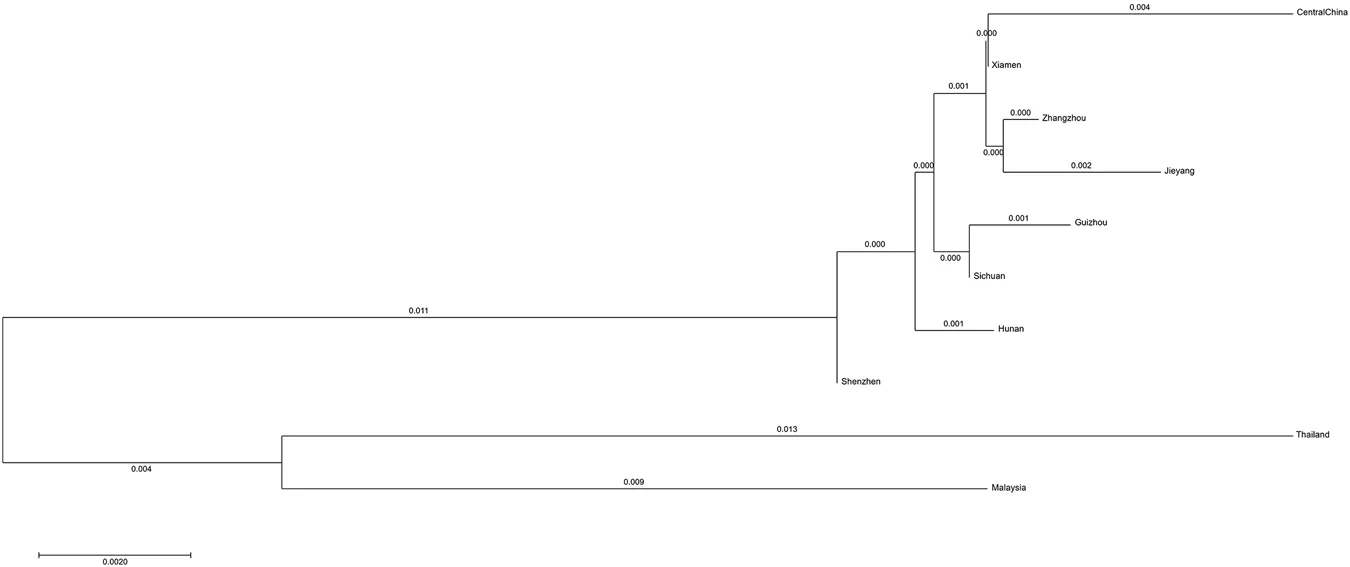
N-J tree for Shenzhen population and reference populations based on 23 STR loci. Phylogenetic tree constructed by using the neighbor-joining (N-J) algorithm. Genetic relationships and population genetic admixture structure among 7 Chinese populations and 2 foreign populations based on the genetic variation of 23 autosomal STRs included in the Huaxia Platinum six-dye amplification system.

**TABLE 6:** PCA (principal component analysis) results based on the allele frequencies of the 23 shared STR loci.

Populations	PC1 (62.15%)	PC2 (20%)
Shenzhen	−0.048424583	0.0203183136578587
Central China	−0.131946629	−0.034468437
Hunan	−0.061030278	0.0157118272259378
Guizhou	−0.068735633	0.0296280765033044
Xiamen	−0.066336024	−0.000988394
Zhangzhou	−0.073305086	0.00189193682688729
Jieyang	−0.066578769	−0.004656586
Sichuan	−0.067880579	0.00834684107065449
Thai	0.319999743871018	0.165280750633617
Malay	0.264237837153498	−0.201064328

**FIGURE 3:**
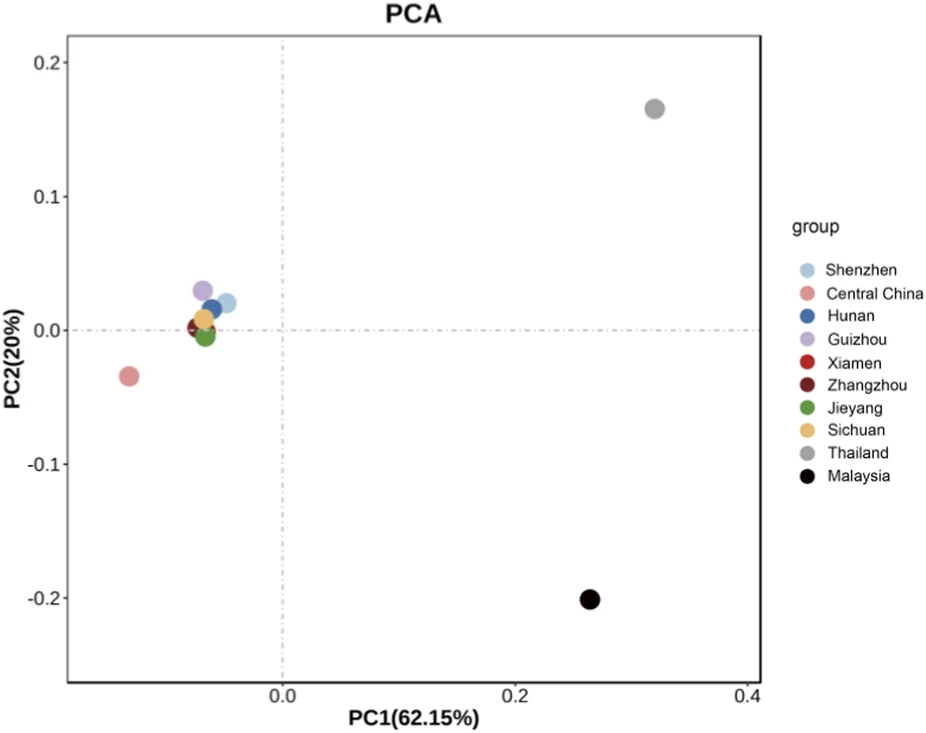
Principal component analysis (PCA) plots of the Shenzhen Han population and 9 other reference Han populations. The first and second principal components account for 62.15% and 20% of the variance, respectively.

Taken together across the two principal components, Shenzhen is positioned at coordinates (−0.048, 0.020), falling entirely within the dense Chinese core cluster. The overall sample distribution clearly displays a three-cluster pattern: a Chinese core cluster (concentrated in the left-middle area), Thailand (upper-right area), and Malaysia (lower-right area).

### Overview of the mutation rate

In this work, 30 mutations were observed at 14 loci in a total of 2,514 parent-child allelic transfers from 1,309 paternity testing cases (Table 7). Mutation counts, allelic transmissions, mutation rates and the 95% CI for each locus are described, 30 mutation events were observed in 2,514 meiosis and referred to 14 of the 23 autosomal STR loci, with an overall mutation rate of 0.0852% (95% CI, 0.10–2.87 × 10^−3^) (Table 8). The locus-specific mutation rates of the 14 STR loci ranged from 0.040% to 0.159%, with the maximum value found at locus D5S818 and no mutation event observed at 9 STR loci (D3S1358, D2S441, TH01, D22S1045, D13S317, D10S1248, D1S1656, D2S1338 and Penta D). Considering mutation steps, twenty-seven of them were one-step mutations, two were two-step mutations and one was a three-step mutation. The proportion of one-step mutations was approximately 90%.

**TABLE 7:** Details of our selected families with confirmed parentage.

Case Types	Parents	Father	Mother
One-child	797	219	156
Two-children	90	16	6
Three-children	16	5	—
Four-children	2	1	—
Five-children	1	—	—
No. of cases	906	241	162
No. of transmissions	2076	270	168

**TABLE 8:** Paternal, maternal, and total number of mutations, mutation rate, and confidence interval for the 23 STR loci.

Locus	Paternal				Maternal				In total			
	No. of meiosis	No. of mutations	Mutation rate (×10^–3^))	95% CI (×10^–3^)	No. of meiosis	No. of mutations	Mutation rate (×10^–3^)	95% CI (×10^–3^)	No. of meiosis	No. of mutations	Mutation rate (×10^–3^)	95% CI (×10^–3^)
D5S818	1,308	2	1.530	0.18–5.52	1,206	2	1.660	0.20–5.99	2,514	4	1.590	0.43–4.07
CSF1PO	1,308	1	0.760	0.02–4.26	1,206	2	1.660	0.20–5.99	2,514	3	1.190	0.25–3.49
D8S1179	1,308	1	0.760	0.02–4.26	1,206	2	1.660	0.20–5.99	2,514	3	1.190	0.25–3.49
FGA	1,308	3	2.290	0.47–6.70	1,206	0	0.000	0.00–3.06	2,514	3	1.190	0.25–3.49
D6S1043	1,308	3	2.290	0.47–6.70	1,206	0	0.000	0.00–3.06	2,514	3	1.190	0.25–3.49
D16S539	1,308	2	1.530	0.18–5.52	1,206	0	0.000	0.00–3.06	2,514	2	0.800	0.10–2.87
D12S391	1,308	2	1.530	0.18–5.52	1,206	0	0.000	0.00–3.06	2,514	2	0.800	0.10–2.87
vWA	1,308	2	1.530	0.18–5.52	1,206	0	0.000	0.00–3.06	2,514	2	0.800	0.10–2.87
Penta E	1,308	1	0.760	0.02–4.26	1,206	1	0.830	0.02–4.62	2,514	2	0.800	0.10–2.87
D18S51	1,308	2	1.530	0.18–5.52	1,206	0	0.000	0.00–3.06	2,514	2	0.800	0.10–2.87
D7S820	1,308	1	0.760	0.02–4.26	1,206	0	0.000	0.00–3.06	2,514	1	0.400	0.01–2.22
TPOX	1,308	1	0.760	0.02–4.26	1,206	0	0.000	0.00–3.06	2,514	1	0.400	0.01–2.22
D19S433	1,308	1	0.760	0.02–4.26	1,206	0	0.000	0.00–3.06	2,514	1	0.400	0.01–2.22
D21S11	1,308	0	0.000	0.00–2.82	1,206	1	0.830	0.02–4.62	2,514	1	0.400	0.01–2.22

CI: exact Binomial CI, using the inverse Fisher F function.

As to the origin of mutation events, a total of 22 paternal and 8 maternal mutations could be determined under 1,308 paternal meiosis and 1,206 maternal meiosis. The overall average paternal and maternal mutation rates were 0.1201% and 0.0474%, respectively. The ratio of paternal versus maternal mutation rate was approximately 2.5:1.

### Sequencing of the STR mutations

We selected samples with STR gene mutations (where the STRs are complex or composite sequences) to sequence and analyze the mutation characteristics, thereby identifying which bases have undergone changes in their repeat numbers. For example, in sample 1, the D21S11 genotype of the mother is (28, 35), and of the child is (32, 34). According to the principle of minimum mutation steps, 35 mutates to 34. From the sequence analysis, the D21S11 sequence of mother is [TCTA]8 [TCTG]5 [TCTA]3 TA [TCTA]3 TCA [TCTA]2 TCCA TA [TCTA]14, while of the child is [TCTA]8 [TCTG]5 [TCTA]3 TA [TCTA]3 TCA [TCTA]2 TCCATA [TCTA]13. It can be seen that the sequence with altered repeat number is TCTA, with the child having one less repeat of this sequence. Addtionally, we selected another 10 sets of samples for sequencing analysis, with the results shown in the Table 9.

**TABLE 9:** Sequence analysis of some mutant samples.

​	​	Mother		Children		Father	
Sample No.	STR loci	Genotype	Sequence	Genotype	Sequence	Genotype	Sequence
Sample 1	D21S11	28	[TCTA]5 [TCTG]6 [TCTA]3 ta [TCTA]3 tca [TCTA]2 tccata [TCTA]9	32	[TCTA]5 [TCTG]6 [TCTA]3 ta [TCTA]3 tca [TCTA]2 tccata [TCTA]13	​	​
​	​	35	[TCTA]8 [TCTG]5 [TCTA]3 TA [TCTA]3 TCA [TCTA]2 TCCA TA [TCTA]14	34	[TCTA]8 [TCTG]5 [TCTA]3 TA [TCTA]3 TCA [TCTA]2 TCCATA [TCTA]13	​	​
Sample 2	D8S1179	13	[TCTA]1 [TCTG]1 [TCTA]11	15	[TCTA]2 [TCTG]1 [TCTA]12	​	​
​	​	16	[TCTA]2 [TCTG]1 [TCTA]13	17	[TCTA]2 [TCTG]1 [TCTA]14	​	​
Sample 3	FGA	18	—	21	[GGAA]2 [GGAG]1 [AAAG]13 [AGAA]1 [AAAA]1 [GAAA]3	22	[GGAA]2 [GGAG]1 [AAAG]14 [AGAA]1 [AAAA]1 [GAAA]3
​	​	21	—	26	[GGAA]2 [GGAG]1 [AAAG]18 [AGAA]1 [AAAA]1 [GAAA]3	25	[GGAA]2 [GGAG]1 [AAAG]17 [AGAA]1 [AAAA]1 [GAAA]3
Sample 4	D12S391	​	​	24	[AGAT]15 [AGAC]8 [AGAT]1	20	[AGAT]13 [AGAC]6 [AGAT]1
​	​	​	​	24	[AGAT]16 [AGAC]7 [AGAT]1	25	[AGAT]16 [AGAC]8 [AGAT]1
Sample 5	VWA	​	​	14	[TAGA]3 TGGA [TAGA]3 [CAGA]4 [TAGA]1 [CAGA]1 [TAGA]1	16	[TAGA]11 [CAGA]4 [TAGA]1
​	​	​	​	20	[TAGA]15 [CAGA]4 [TAGA]1	21	[TAGA]16 [CAGA]4 [TAGA]1
Sample 6	D8S1179	12	[TCTA]12	14	[TCTA]1 [TCTG]1 [TCTA]12	​	​
Sample 7	FGA	​	​	21	[GGAA]2 [GGAG]1 [AAAG]13 [AGAA]1 [AAAA]1 [GAAA]3	23	[GGAA]2 [GGAG]1 [AAAG]15 [AGAA]1 [AAAA]1 [GAAA]3
Sample 8	FGA	23	—	23	[GGAA]2 [GGAG]1 [AAAG]15 [AGAA]1 [AAAA]1 [GAAA]3	22, 24	[GGAA]2 [GGAG]1 [AAAG]14 [AGAA]1 [AAAA]1 [GAAA]3
Sample 9	D8S1179	​	​	13	[TCTA]1 [TCTG]1 [TCTA]11	12	[TCTA]12
​	​	​	​	16	[TCTA]2 [TCTG]1 [TCTA]13	17	[TCTA]2 [TCTG]1 [TCTA]14
​	​	​	​	17	[TAGA]12 [CAGA]4 [TAGA]1	16	[TAGA]11 [CAGA]4 [TAGA]1
Sample 10	VWA	14	—	21	[TAGA]15 [CAGA]5 [TAGA]1	20	[TAGA]14 [CAGA]5 [TAGA]1
​	​	​	​	14	[ATCT]14	18	[ATCT]5 [ATGT]1 [ATCT]12
Sample 11	D6S1043	​	​	21	[ATCT]5 [ATGT]1 [ATCT]15	20	[ATCT]5 [ATGT]1 [ATCT]14

## Discussion

Our study provides what we believe to be the first published autosomal STR data for the general Shenzhen Han population using 23 loci genotyped using the Huaxia Platinum System. We analyzed the genetic Han population data from Shenzhen and presented the comprehensive population comparisons. Forensic parameters obtained from the present study demonstrate the suitability of the 23 autosomal STR loci tested for use in forensic and paternity analysis. Genetic structure investigation among 1,226 Chinese individuals from Shenzhen and other 9 populations based on Huaxia Platinum panel revealed that genetic differences are observed among different geographical location. Genetic distance reveals that Shenzhen Han population is more closely related to Hunan Han Chinese populations than any other reference populations. This observation reflects cultural similarities and geographical factors, coupled with the long history of migration and trading activities between Shenzhen and Hunan. We believe our new autosomal STR data from this study are not only of direct practical relevance for forensic science but also for population genetic studies.

In our study, mutations at 14 STR loci from 1,309 paternity test cases were counted and analyzed, and a total of 30 mutations were observed with an average mutation rate of 0.0852%. Mutation rates of frequently used autosomal STR loci were reported to be in the range of 0.1%–0.2% (Brinkmann et al., 1998),while our results showed that the mutation rates of the 14 STR loci were in the range of 0.000%–0.159%, with the maximum value found at locus D5S818. The evaluation of mutation rates of STR loci can be affected by sampling populations and population configurations. Due to the small sample size and few mutation cases, the calculated mutation rate may be biased. In this study, there were 403 duo-cases among 1,309 cases, some potential mutation events might be neglected if either of the parents was absent, which will result in underestimated mutation rates in the population. For example, in one of our paternity testing cases, the mother’s genotype at the FGA locus is 23, the child’s genotype is 19,23, and the alleged father’s genotype is 18,23. If analyzed as a trio, the mother transmits the 23 allele, while the father’s 18 allele mutated to 19 and be passed on to the child, indicating a paternal one-step mutation. However, if the mother-child and father-child pairs are evaluated separately as duos, the child’s genotype complies with Mendelian inheritance, and no mutation is observed. This discrepancy would lead to a bias in the estimation of mutation rates. Thus, larger sample sizes were needed to obtain meaningful mutation rates.

Besides, a limitation of this work is that the mutation detection in our study was based on a single-tube multiplex kit, while potential allelic dropout or primer-binding site mutations cannot be excluded. Further additional confirmatory experiments will be necessary to address this limitation such as re-amplification with alternative primers or deep sequencing of anomalous alleles.

The majority of mutations in our study were one-step mutations, accounting for 90% of instances, in agreement with the stepwise model for STR mutation which demonstrated that single-step mutation could be frequently observed compared with multi-steps mutations (Brinkmann et al., 1998). Apart from the vast majority of single-step mutations, rare multi-step mutations can be observed. Therefore, the interpretation of mutational events should not rigidly be restricted to the shortest possible mutational step, because rare but true multi-step mutations can easily be overlooked, if haplotype analysis is not possible (Dauber et al., 2012).

Previous study found that mutations from fathers could be higher than that of originated from mothers (Brinkmann et al., 1998). A possible explanation was that mutation events in the male germline were more frequent than in the female germline (Lu et al., 2012; Jin et al., 2016), the male spermatocytes underwent more cell divisions and DNA replications than female oocytes did during meiosis (Brinkmann et al., 1998). In our study, the present ratio of mutation origin was 2.5:1 (paternal origin versus maternal origin), which also supported the conclusion to a certain extent. Additionally, the paternal age distribution and sexual ratio in our study may have influenced the estimation of the overall average mutation rate to some extent. For example, as shown in the Table 7, the total number of cases for which paternal mutation rates could be calculated was 1,147 (including 906 trios and 241 duos), while the maternal cases totaled 1,068 (including 906 trios and 162 duos). The paternal-to-maternal ratio was 1.07, indicating a slightly higher proportion of paternal cases. If only duos were considered, the paternal-to-maternal ratio increased to 1.49 (241/162). Given that paternal mutations are more prone to occur, the overall mutation rate calculated in this study may be slightly skewed. Moreover, since increased paternal age is associated with a higher mutation rate (Huang et al., 2021), it can be speculated that in paternity identity cases involving elderly individuals, due to the increased likelihood of mutations, genotyping abnormalities other than mutations may occur, such as loss of heterozygosity, imbalanced amplification, and so on. In our study, we observed a difference in the parental age distribution among the mutation cases. The paternal age distribution (n = 22) was relatively broad, with cases spanning from the 20–30 age group to over 50 years, and a peak in the 30–40 age group (8 cases). In contrast, the maternal age distribution (n = 8) was more restricted, with all cases occurring between the ages of 20 and 40, and the majority (5 cases) concentrated in the 20–30 age group. No mutation cases were recorded for parents under 20 years of age in either sex (Table 10). These findings suggest that advanced paternal age may contribute to a wider age range of mutations, whereas maternal mutations appear to cluster within the reproductive peak years. Thus, further investigations with a larger sample size and broader age coverage across different age groups will be necessary to minimize the statistical bias in the mutation rate estimates.

**TABLE 10:** Paternal and maternal age distribution in mutation cases.

Age (years)	Father	Mother
<20	0	0
20–30	7	5
30–40	8	2
40–50	6	1
>50	1	0
In total cases	22	8

Since STR mutation plays essential role in a wide range of applications, especially in the parental testing area of forensic science, we conclude that our study can provide useful mutation information for STR loci included in the Huaxia Platinum System kit and further perfect the usage of the kit in the future.

## Conclusion

In our study, we genotyped 1,226 unrelated healthy individuals from Shenzhen, using the Huaxia Platinum PCR kit to obtain allele frequencies for 23 autosomal STR loci. No significant deviations from HWE were detected after Bonferroni correction and forensic parameters were obtained. Data showed 23 autosomal STRs included in Huaxia Platinum System are informative and polymorphic in Han groups. Population comparisons revealed a close genetic affinity between the Shenzhen Han population and the other Chinese groups, particularly the Hunan Han population.

We randomly selected 906 trios and 403 duos among 1,309 paternity cases with a combined paternity index ≧ 10,000. 30 mutation events occurred at 23 STR loci of the panel during 2,514 meiosis, and the locus-specific mutation rates ranged from 0.000% to 0.159% (D5S818), with an overall mutation rate of 0.0852% (95%CI, 0.10–2.87 × 10^−3^). In summary, our results indicate that the 23 autosomal STR loci exhibit high polymorphism and strong informativeness within the Shenzhen Han population studied. These findings offer valuable insights for future research both forensic and population genetics, and thus represent a robust tool for forensic applications.

## Data Availability

The original contributions presented in the study are included in the article/Supplementary Material, further inquiries can be directed to the corresponding author.
